# Cluster analyses of the TCGA and a TMA dataset using the coexpression of HSP27 and CRYAB improves alignment with clinical-pathological parameters of breast cancer and suggests different epichaperome influences for each sHSP

**DOI:** 10.1007/s12192-022-01258-0

**Published:** 2022-03-02

**Authors:** Philip R. Quinlan, Grazziela Figeuredo, Nigel Mongan, Lee B. Jordan, Susan E. Bray, Roman Sreseli, Alison Ashfield, Jurgen Mitsch, Paul van den Ijssel, Alastair M. Thompson, Roy A. Quinlan

**Affiliations:** 1 Digital Research Service, University of Nottingham, Nottingham, NG8 1BB UK; 2 Dundee Cancer Centre, Ninewells Hospital and Medical School, Dundee, DD1 9SY UK; 3 School of Medicine, University of Nottingham, Nottingham, NG7 2UH UK; 4 School of Computer Science, University of Nottingham, Nottingham, NG8 1BB UK; 5 Faculty of Medicine and Health Sciences, Biodiscovery Institute University Park, Nottingham, NG7 2RD UK; 6 NHS Tayside, Department of Pathology, Ninewells Hospital and Medical School, University of Dundee, Dundee, DD1 9SY UK; 7 Tayside Tissue Bank Ninewells Hospital and Medical School, University of Dundee, Dundee, DD1 9SY UK; 8 Lelystad, Netherlands; 9 Dan L Duncan Comprehensive Cancer Center, Houston, TX 77030 USA; 10 Department of Biosciences, The University of Durham, Upper Mountjoy Science Site South Road, Durham, DH1 3LE UK

**Keywords:** Small heat shock protein, HSP27, HSPB1, Monoclonal antibody specific to phosphorylated Serine 82 in HSP27, Alphab-crystallin, CryAB, HSPB5, Breast cancer, Cluster analysis, Cancer Genome Atlas, Epichaperome, TP53, Estrogen receptor (ER), Progesterone receptor (PR), Patient survival

## Abstract

Our cluster analysis of the Cancer Genome Atlas for co-expression of HSP27 and CRYAB in breast cancer patients identified three patient groups based on their expression level combination (high HSP27 + low CRYAB; low HSP27 + high CRYAB; similar HSP27 + CRYAB). Our analyses also suggest that there is a statistically significant inverse relationship between HSP27 and CRYAB and known clinicopathological markers in breast cancer. Screening an unbiased 248 breast cancer patient tissue microarray (TMA) for the protein expression of HSP27 and phosphorylated HSP27 (HSP27-82pS) with CRYAB also identified three patient groups based on HSP27 and CRYAB expression levels. TMA24 also had recorded clinical-pathological parameters, such as ER and PR receptor status, patient survival, and *TP53* mutation status. High HSP27 protein levels were significant with ER and PR expression. HSP27-82pS associated with the best patient survival (Log Rank test). High CRYAB expression in combination with wild-type *TP53* was significant for patient survival, but a different patient outcome was observed when mutant *TP53* was combined with high CRYAB expression. Our data suggest that HSP27 and CRYAB have different epichaperome influences in breast cancer, but more importantly evidence the value of a cluster analysis that considers their coexpression. Our approach can deliver convergence for archival datasets as well as those from recent treatment and patient cohorts and can align HSP27 and CRYAB expression to important clinical-pathological features of breast cancer.

## Introduction

AlphaB-crystallin (CRYAB, HSPB5) and HSP27 are both members of the small heat shock protein family (Kappé et al. 2010) and each are implicated in the progression of breast cancer (Caporossi et al. 2021; Choi et al. 2019; Wang et al. 2020). Both sHSPs are considered potential targets for the treatment of breast cancer (Caporossi et al. 2021; Lang et al. 2019; Wang et al. 2020). Their individual contributions to the breast cancer chaperome and epichaperome networks have been investigated (Rodina et al. 2016; Wang et al. 2019; Yan et al. 2020). The coassembly of HSP27 and CRYAB has so far not been considered as an important variable in the transcriptional and omic analyses of breast cancer cohorts (Buttacavoli et al. 2021; Klimczak et al. 2019; Zoppino et al. 2018). These previous studies compared expression levels between cancerous and non-cancerous samples to identify the same basic characteristic for the different breast cancer groups represented in the TCGA cohort, namely raised HSP27 expression and lowered CRYAB expression (Buttacavoli et al. 2021; Klimczak et al. 2019; Zoppino et al. 2018). Our study specifically looked at the inter-relationship between both sHSPs as they coassemble when present in the same cell (Mymrikov et al. 2020; Shatov et al. 2020; Zantema et al. 1992). This will affect both client protein selection and will therefore the breast cancer epichaperome (Arrigo 2013; Arrigo and Gibert 2013).

HSP27 expression correlates with estrogen receptor (ER) expression (Andersen et al. 1989; Damstrup et al. 1992; Takahashi et al. 1995) and estrogen itself regulates HSP27 expression (Dunn et al. 1993). HSP27 is a key component in the trafficking of steroid receptors, including ER and progesterone receptor (PR) to the plasma membrane (Razandi et al. 2010). HSP27 binds to both the ER (Chen et al. 2004) and human epidermal growth factor receptor 2 (HER2; (Kang et al. 2008)) to stabilize them. Estrogen receptor (ER) status is used in classifying breast cancer (Heng et al. 2017; Søkilde et al. 2019) as well as for prognosis and treatment eg (Gu et al. 2020; López-Sánchez et al. 2020; Reis-Filho and Pusztai 2011). The availability of the National Cancer Institute Cancer Genome Atlas has facilitated these investigations (Chierici et al. 2020; Kan et al. 2018; Li et al. 2020) and has been used to assess the contribution of individual chaperones on the basis of their normalized expression levels and then identify potential gene networks important in breast cancer (Buttacavoli et al. 2021; Klimczak et al. 2019; Zoppino et al. 2018).

CRYAB is expressed in the basal cells of normal ductal epithelium, but its expression is robustly elevated in breast cancer as detected by immunohistochemistry (IHC) methods (Kabbage et al. 2012; Moyano et al. 2006) and in the rare, but aggressive, metaplastic subtype of triple negative breast carcinomas (TNBC; ER^−^/PR^−^ HER2^−^; (Sitterding et al. 2008)) that metastasize (Koletsa et al. 2014; Malin et al. 2016; Voduc et al. 2015). CRYAB has been reported to be a marker of poor prognosis in breast cancer (Ivanov et al. 2008; Moyano et al. 2006; Voduc et al. 2015) and has oncogenic potential (Moyano et al. 2006), although this is open to discussion (Zoppino et al. 2018). A recent study identified *CRYAB* as part of a gene cluster associated with good prognosis despite its significant association with the clinical-pathological parameters of *ER*^*−*^*/PR*^*−*^ and *HER2*^*−*^ (Buttacavoli et al. 2021), a breast cancer patient group with poor prognosis (Parker et al. 2009).

The same multi-omic analysis (Buttacavoli et al. 2021) identified *HSP27* as part of a gene network associated with poor prognosis. It also found *HSP27* to be significantly associated with both estrogen (*ER*) and progesterone (*PG*) receptor expression (Buttacavoli et al. 2021) and yet both receptors are characteristic of luminal A patients (Cancer Genome Atlas Network 2012; Zoppino et al. 2018), a group with the best patient survival (Parker et al. 2009). It is also appreciated that the same chaperone can be associated with both good and poor patient survival depending on the cancer type demonstrating that context is key (Klimczak et al. 2019), but for HSP27, like CRYAB, that context remains unclear.

Recently, more focus has been given to HSP27 phosphorylation in breast cancer (Buttacavoli et al. 2021; Wang et al. 2020). HSP27 is phosphorylated at serine 82 by both Akt1/2 (Rane et al. 2003), MAKAPK2 (MK2;(Stokoe et al. 1992)) and by p38a (MAPK14;(Eyers et al. 1999)). It has been reported that HER2 suppresses the p38 pathway via Skp2-mediated degradation in a mechanism that involves Akt (Wang et al. 2020). The HER2 pathway is associated with HSP27-15pS and HSP27-78pS rather than HSP27-82pS (Hwang et al. 2020; Zhang et al. 2007). Monitoring HSP27-82pS could therefore be a useful indicator of the activity of the p38/MK2/Akt complex (Zheng et al. 2006). The availability of a monoclonal antibody specific to this phosphorylated site in HSP27 and compatible with IHC analyses would therefore assist future studies.

Here we use cluster analysis to assess the relative and joint expression of *CRYAB* and *HSP27* associated with breast cancer in the Cancer Genome Atlas. This targeted approach identified three different patient populations based upon their combined HSP27 and CRYAB expression levels within the breast cancer samples. This observation is replicated in an unbiased primary breast cancer patient cohort using a tissue microarray (TMA) comprising samples from 248 patients with full clinical and pathology data including patient survival. We generated and characterized a monoclonal antibody specific to the phosphorylation site on serine 82 in HSP27 and report that both HSP27 and HSP27-82pS expression are significant (Log Rank (LR)) for patient survival. Statistical analyses of our data suggest that CRYAB and HSP27 both contribute to breast cancer, but their epichaperome influences are different.

## Materials and methods

### Cluster analysis of the TCGA dataset

The Cancer Genome Atlas (TCGA; https://www.cancer.gov/about-nci/organization/ccg/research/structural-genomics/tcga) was subjected to consensus clustering analysis using the following methodology (Soria et al. 2010) to select the number of clusters so the similarity between the clusters is minimised. Two clustering methodologies were applied, K-Means and Partition Around Medioids (PAM) clustering to increase robustness instead of relying on a single clustering algorithm’s assignment. The output from both clustering approaches are compared to understand where the different approaches agree and disagree on the cluster memberships for all the data samples. The data were formatted appropriately before cluster analysis was attempted. When raw data is clustered with variables that have values of vastly different magnitudes, then the clustering result is likely to be biased and the cluster designations will be influenced most by the variable that has the largest numerical values compared to the others. To overcome this, a normalization process was applied. The raw data was log transformed and then Z-standardized to eliminate the unwanted influence of differences in magnitude and skewness of the raw data (Mohammad and Usman 2013).

### Tissue microarray construction

The cohort used in this study (TMA24) has been reported previously (King et al. 2012) and comprises unselected women with primary, previously untreated breast cancer, who attended clinic and were treated at Tayside University Hospitals, Scotland from 1997 to 2002. Samples were obtained at the time of surgery and only where sufficient cancer material was available. Ethics approval was given by the Tayside Tissue Bank for the use of these samples (Letter included) under delegated responsibility from the Tayside Research and Ethics Committee (Ref. 07/S1402/90). A few samples in TMA24 were obtained from patients before it was a legal requirement to obtain consent. Access to the clinical records was granted via the protocols in place with the local Caldicott Guardian.

### Immunohistochemical staining and TMA scoring

Sections from the TMA block were processed and scored as previously described (Detre et al. 1995). In total, 207, 208, and 204 cases were available for scoring for HSP27, HSP27-82pS and CRYAB respectively (see Table 1 for antibody details). Unless otherwise stated, the cut-point (0–3 = low levels; 4–18 = high levels) was used to score staining and to be consistent with previous studies on HSP27 (Love and King 1994; Thanner et al. 2005). The primary observer was blinded to sample identity, with observer error moderated by a second observer equally blinded to sample identity. *TP53* mutation status was determined as described previously (King et al. 2012).

**Table 1:** Summary of the antibodies and IHC details used in this study

Specificity	Type	Source	Clone	Dilution	Antigen Retrieval	Serum Block
Anti-CRYAB	Mouse Monoclonal	Chicken lens	2D2B6 (Sawada et al. 1993)	1–100	Citric Acid	Horse
Anti-HSP27 ERD5	Mouse Monoclonal	MCF7 cells	ERD-5 (Love and King 1994)	1–4000	Citric Acid	Horse
Anti-HSP27- 82pS	Mouse Monoclonal	Synthetic phospho-peptide	1.2 (Gorog et al. 2009; Martin et al. 2001; Srinivasan et al. 2008)	1–25	Citric Acid	Horse

#### Characterization of the HSP27-82pS monoclonal antibody

A monoclonal antibody was generated to the peptide LSRQLS^82^SGVSEC, phosphorylated at serine 82. It was coupled via a C-terminal cysteine to keyhole limpet haemocyanin as described (Eyers et al. 1999). The corresponding non-phosphorylated peptide (LSRQLSSGVSEC) as well as phosphorylated and non-phosphorylated peptides for the Ser15 (LLRGPS^15^WDPFRC) and Ser78 (YSRALS^78^RQLSSC) sites were used to confirm site specificity in a slot blot approach using a whole cell extract of the breast cancer cell line MCF7 cells. Several human (MCF7, U373MG) and animal (CHO, BHK21, MDCK) tissue culture cell lines were used to prepare whole cell extracts for immunoblotting as described (van den Ijssel et al. 2003; van den IJssel et al. 1998). Samples from bovine lens and rat heart were also included to demonstrate the broad cross reactivity expected from HSP27 sequence alignments and the specificity of the monoclonal antibody clone 1.2. In some cases, cells were heat shocked at 42^0^C (1 h) or treated with sodium arsenite (100 µM) for 2 h. Recombinant, unphosphorylated human HSP27 was produced in *E.coli* and purified as described (Perng et al. 1999) and was used as a negative control. These studies compliment those where the inhibitor SB203580 was used to evidence specificity (Gorog et al. 2009; Martin et al. 2001; Srinivasan et al. 2008). Lastly, the ability of the antibody to recognize the phosphorylated epitope in an archival cervical intra-epithelial neoplasia sample (data not shown) and a metastatic breast cancer sample was confirmed by immunohistochemistry.

#### Testing for significance

All data within the project were then analyzed using either the Fishers Exact Test (FET) for categorical data, independent *t-*test for continuous data, with all reported *p* values being two tailed or for survival analyses the Kaplan Meier method was used with the Log Rank (LR) method to test for significance. SPSS (Version 27) was used for all analyses to test significance across TMA24 and clustering data. Results were considered significant at an α level of 5% (*p *≤ 0.05).

## Results

### Cluster analysis of the TCGA breast cancer dataset

**Fig. 1:**
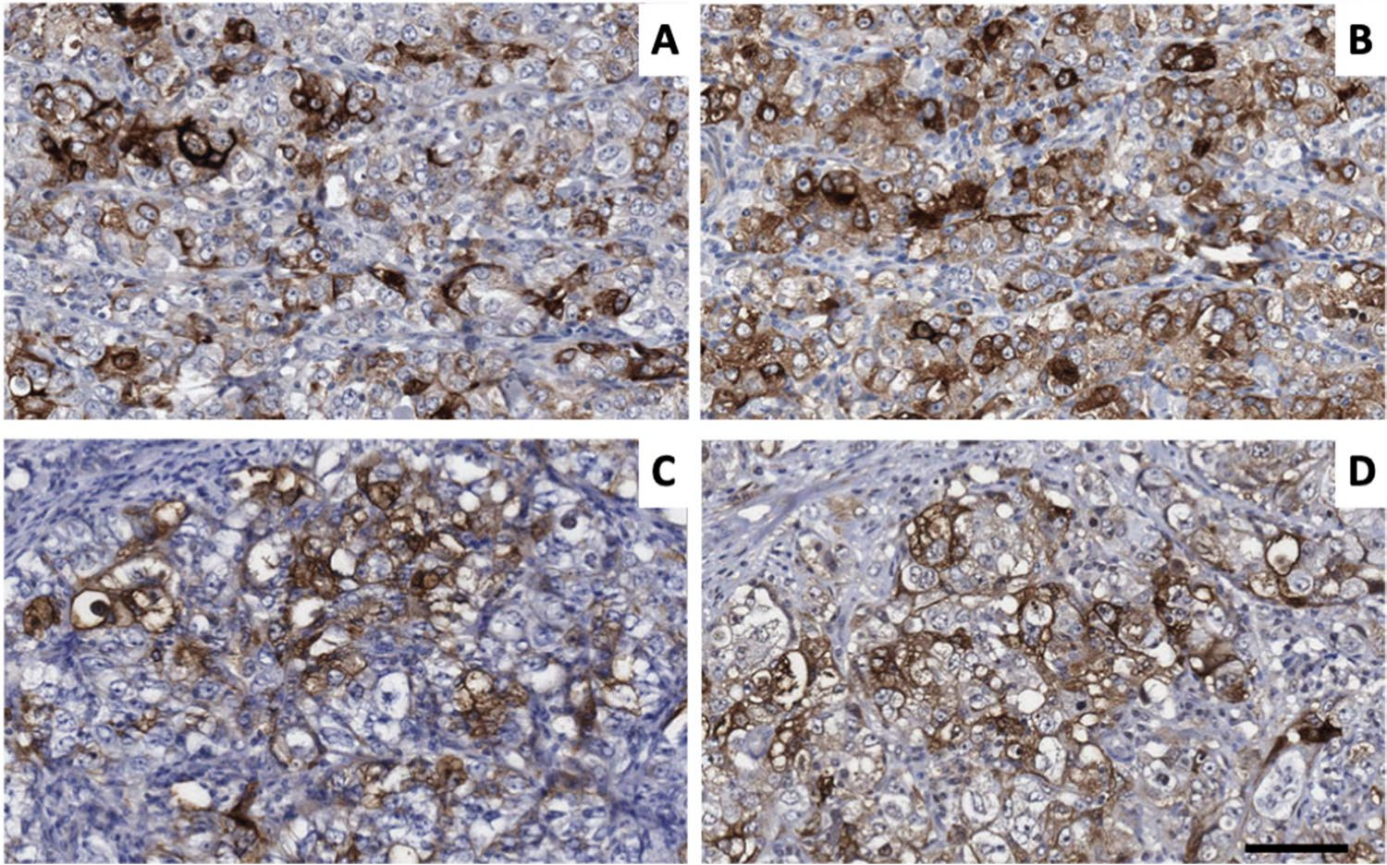
**Robust staining of triple negative invasive ductal breast carcinoma samples with both HSP27 and CRYAB antibodies**. Pairs of consecutive sections (**A** and **B**; **C** and **D**) from two separate triple negative (ER^−^, PR^−^, Her2^−^) cases of invasive ductal carcinoma of the breast were stained with CRYAB and HSP27 antibodies. The following antibody combinations were used: **A**: Anti-CRYAB, Riken mouse monoclonal 2D2B6. **B**: Anti-HSP27, Cancer Research Technology, mouse monoclonal ERD5. **C**: Anti-CRYAB, Abcam mouse monoclonal 1b6.1-3G4 (ab13496). **D**: Anti-HSP27, Abcam rabbit polyclonal (Ab47436). Bar = 100 µm.

Previous studies (Buttacavoli et al. 2021; Klimczak et al. 2019; Zoppino et al. 2018) investigated *HSP* expression in breast cancer samples using TCGA, MET500, CPTAC (Chandrashekar et al. 2017) and METABRIC (https://ega-archive.org/dacs/EGAC00001000484) databases plus associated tools (eg UALCAN; (Chandrashekar et al. 2017)). The clinical parameters used by these three recent studies were not identical, but all three correlated *HSP* expression patterns with patient survival. All three report that *HSP27* transcripts were upregulated and that *CRYAB* transcripts were strongly downregulated. We confirmed that both CRYAB and HSP27 can be robustly expressed in such breast cancer cases as shown in tissue sections (Fig. 1).

**Fig. 2:**
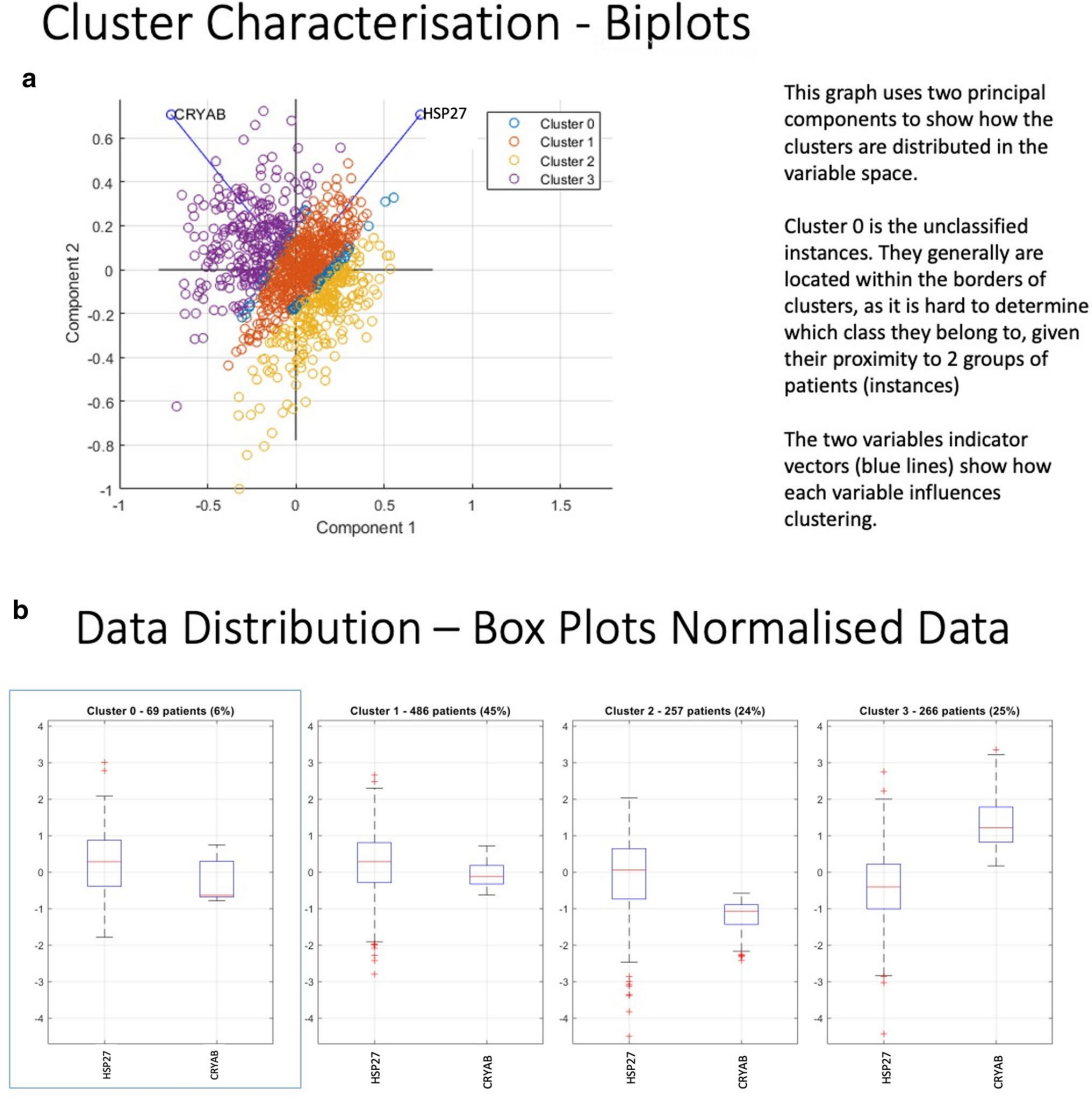
**Cluster Analysis of the breast cancer dataset in the TCGA. A**. Biplot of a two principal component analysis of the breast cancer cohort in the TCGA dataset showing the distribution in variable space. The principal components are a non-orthogonal transformation of the two variables, HSP27 and CRYAB expression levels respectively. Cluster 0 comprises 69 patients that were unassigned to one of the three other clusters, which were separated on the basis of HSP27 and CRYAB expression levels. **B**. Three clusters based upon the expression of HSP27 and CRYAB were identified for the breast cancer cohort within the TCGA dataset. The relative log and z-transformed values in the box-whisker plots show the relative changes in expresison compared to a control group. Cluster 1 is respresented by 45% of the TCGA breast cancer cohort and is the cluster where relative levels of HSP27 are increased. In contrast, CRYAB expression is increased in cluster 3.

We clustered the breast cancer dataset within TCGA (Cancer Genome Atlas Network 2012) with respect to *HSP27* and *CRYAB* expression. Validity indexes were calculated based on both k-Means and PAM clustering. Applying both algorithms to the TCGA dataset and then overlapping the results of both to examine the agreement between the two methodologies yielded three clusters with a very high overall agreement with a К score of 0.91 (Fig. 2). The biplot of the cluster analysis evidence three clusters and show that both *HSP27* and *CRYAB* influence the clustering result (Fig. 2A). Only 69 of the 1078 data points were unassigned to one of these three clusters because both algorithms could not agree on their group membership (Fig. 2B) and these were found at the cluster borders (Fig. 2A). Cluster 1 is the largest, comprising 45% of all data points, whilst clusters 2 and 3 have similar sizes (24% and 25% respectively; Fig. 2B). When looking at the relative z-transformed log values in the box plots, the distinction between the three clusters is quite clear. This identifies one cluster (Cluster 3) where *CRYAB* levels are elevated compared to the others, another where *CRYAB* levels are reduced (Cluster 2) and lastly one cluster where *HSP27* levels are raised (Cluster 1). These data suggest that breast cancer patient groups can be distinguished based on the expression of *CRYAB* and *HSP27*, but how this relates to disease outcome requires further investigation and the availability of greater longitudinal patient data.

## HSP27, HSP27-82pS, and CRYAB within breast cancer subgroups

**Fig. 3:**
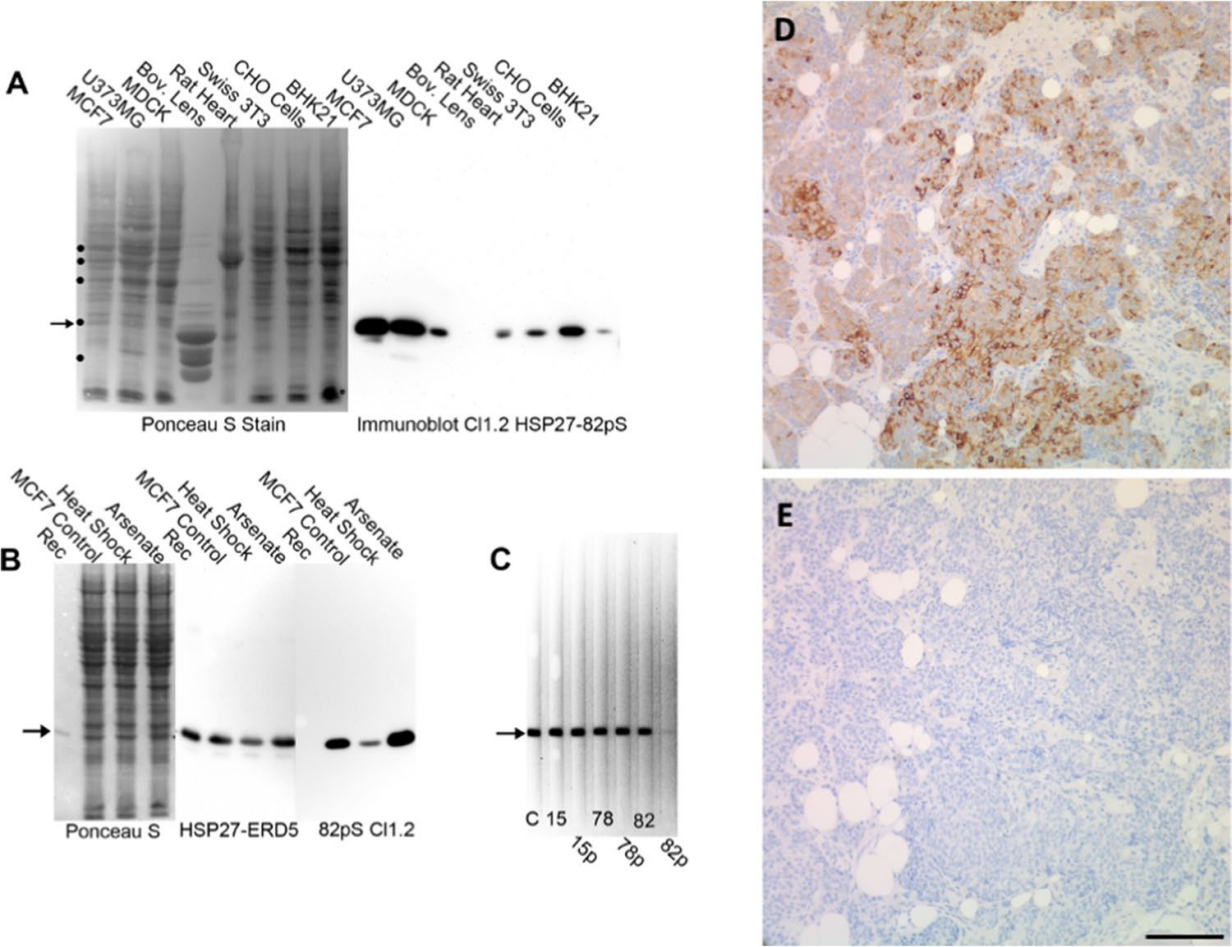
**Specificity of the HSP27-82pS monoclonal antibody.** **A**. Tissue culture cell (MCF7, U343MG, MDCK, Swiss 3T3, CHO and BHK21) and tissue (bovine lens, rat heart) extracts were separated by SDS-PAGE, transferred to nitrocellulose and stained with Ponceau S to verify protein transfer. Immunoblotting demonstrated excellent cross-reactivty and specificity for HSP27-82pS (arrow). All but the bovine lens extract produced a positive immunoreactive band. No cross-reaction with CRYAB was seen in either the U373MG or the bovine lens sample. MCF cells were subjected to a sublethal heat shock (420C for 1 h) or exposed to 100 µM arsenate for 2 h, before extracting total protein. Molecular weight markers (•) in order of increasing relative electrophoretic mobility are 54, 50, 46, 27, and 22 kDa. B. Specificity of clone 1.2 for HSP27-82pS in extracts from the breast cancer cell line MCF7. MCF7 cells were heat shocked and exposed to arsenite to alter the HSP27 phosphorylation. Recombinant HSP27 was included as a negative control. Protein and cell extract were separated by SDS-PAGE, transferred to nitrocellulose (Ponceau S) and then probed with anti-HSP27 and the clone 1.2 that was raised against HSP27-82pS. Notice the recombinant HSP27 was not recognised, but an immunoreactive band was detected in the MCF7 samples. **C**. Site specificity of the HSP27-82pS monocolonal 1.2. A whole cell extract from MCF7 cells was used for a slot blot assay. Phosphorylated and unphosphorylated HSP27 peptides were added to the clone 1.2 HSP27-82pS antibody mixture as indicated. Peptide addition made no difference to the detection of an immunop[ositicvve band, except for the lane where the HSP27-82pS peptide had been included. These data confirm the specificity of the antibody for phosphorylated serine 82 in HSP27. **D**. Immunohistochemical staining with the monoclonal clone 1.2 HSP27-82pS of an invasive ductal carcinoma of the breast that has become metastatic and spread to the femur. The tissue culture supernatant from the 1.2 clone was diluted 50 fold. There are widespread regions of intense staining within the metastic tissue evidencing the utility of this antibody for immunohistologiocal analyses. **E**. Negative control from a serial section showing the lack of positive staining when the monoclonal HSP27-82pS antibodies are omitted. Scale bar = 100 µm

We selected a tissue microarray (TMA24) that has been studied previously (King et al. 2012) to assess and build on our cluster analysis. Germaine to our study, TMA24 comprises unselected women with primary, previously untreated breast cancer, who attended clinic and were treated from 1997 to 2002. It had also been assessed for *TP53* mutation status given that a quarter of breast cancers express mutant TP53 (Olivier et al. 2010). We also generated a monoclonal antibody to detect HSP27 phosphorylated at serine 82 (HSP27-82pS) as an important p38 pathway target (Wang et al. 2020) in order to stratify further the HSP27 expression data given that relative changes for *HSP27* were not as great as for *CRYAB* as shown by our cluster analysis (Fig. 2B). The specificity of the HSP27-82pS monoclonal antibody was confirmed using extracts from a panel of cell and tissue extracts and from heat shocked and arsenite treated MCF7 cells before using the antibodies to detect the phosphorylated epitope in archival tissue samples by standard IHC (Fig. 3).

**Fig. 4:**
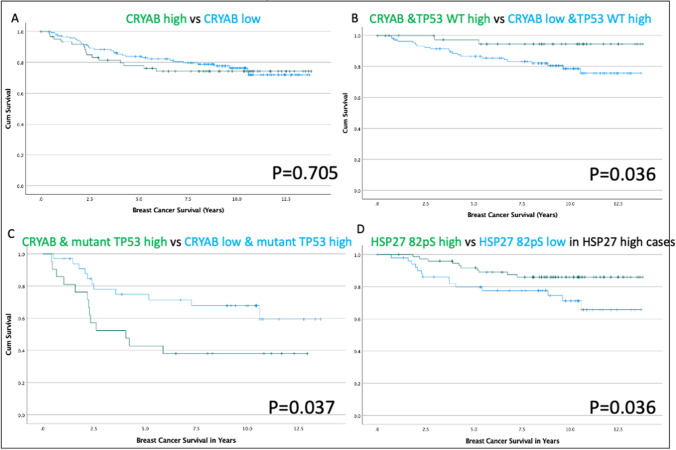
**Association of CRYAB, HSP27, HSP27-82pS with survival for breast cancer patients based on an analysis of the TMA24 dataset**. The association of CRYAB with breast cancer survival within the *TP53* WT (A) and *TP53* mutant (B) subgroups. The association of HSP27 (C) and HS27-82pS (D) with ER and PR expression. The LR score is included on each panel.

Table 2 is a summary of the significance of the associations between HSP27, CRYAB, and key clinical-pathological breast cancer parameters. A significant inverse relationship (high HSP27, low CRYAB expression) between these two sHSPs was seen for patients that were ER and PR positive (Fishers Exact Test (FET) *p* =  ≤ 0.001), suggestive of an association between steroid receptor maturation and HSP27 expression. HSP27 was also significantly associated with the expression of wild type TP53 and conversely CRYAB was significantly associated with mutant TP53 expression (Table 2). We find similar associations in our initial analysis of the TMA24 data. Neither low nor high CRYAB expression were significantly associated with survival in the whole TMA24 patient group. High CRYAB expression was significantly associated with patient survival when coexpressed with high levels of wild-type *TP53* (Fig. 4B). High levels of both CRYAB and mutant TP53 significantly associated with poor patient survival (Fig. 4C) and low CRYAB expression with high mutant TP53 associated with better patient survival. There was, however, a statistically significant association between high HSP27-82pS expression and patient survival in the high HSP27 expressing patients of the TMA24 cohort (Fig. 4D) demonstrating the advantage of screening for HSP27 82pS rather than HSP27 levels alone. This is the first time that HSP27-82pS has been investigated by IHC in a TMA of unbiased breast cancer patients. From these data it is apparent that there are differences in terms of patient survival and patient groups depending on the expression of ER, PR, and *TP53* mutation status in combination with HSP27 and CRYAB expression.

**Table 2:** Summary of potential associations for the TCGA and TMA24 datasets

HSP27 and CRYAB Datasets	ER Neg	ER Pos	PR Neg	PR Pos	*TP53* WT	*TP53* Mutant
TCGA: *HSP27* High	35	418	94	359	322	104
TCGA: *CRYAB* High	126	146	149	122	118	123
TCGA: *HSP27* & *CRYAB* Low	64	183	82	163	149	88
		*p* < 0.001		*p* < 0.001		*p* < 0.001
TMA: HSP27 High	7	85	25	67	70	23
TMA: CRYAB High	16	8	18	6	11	13
TMA: HSP27 & CRYAB Low	15	27	24	18	34	10
TMA: HSP27 & CRYAB High	12	25	22	15	29	8
		*p* < 0.001		*p* < 0.001		*p* < 0.017
	*ER* Neg	*ER* Pos	*PR* Neg	*PR* Pos	*TP53* WT	*TP53* Mutant
*HSP27* (transformed)	-0.54	0.146	-0.335	0.153	0.138	-0.276
*CRYAB* (transformed)	0.774	-0.228	0.44	-0.22	-0.206	0.322
	All results are significant at *p* < 0.001					

This table provides a breakdown of all comparisons and their statistical significance of ER, PR and TP53 mutation status considered for our patient cohort with respect to the expression of CRYAB and HSP27. Raw TCGA data were also log transformed and then Z-standardized (HSP27 (transformed); CRYAB (transformed)) before being tested again for significance.

## Discussion

Our cluster analysis of the TCGA breast cancer dataset established that there were three different groups—one with low *HSP27* and high *CRYAB* expression (cluster 3), another with reduced *CRYAB* expression, but maintained *HSP27* expression (cluster 2) and the largest that had unchanged *HSP27* and *CRYAB* relative expression levels (cluster 1, Fig. 2B). It is difficult to unequivocally map these clusters onto the data we obtained from the unbiased breast cancer patient group represented in the TMA24, but the cluster analysis shows that the two sHSPs are not independent of each other when it comes to differentiating breast cancer patient groups and appear to show a statistically significant inverse relationship (Table 2).

Our results are consistent with previous studies that demonstrate high *HSP27* expression is linked to *ER* and *PR* expression and high *CRYAB* is linked to basal and triple negative breast cancer (TNBC; ER^-^/ PR^−^/HER2^−^; (Buttacavoli et al. 2021; Parker et al. 2009; Zoppino et al. 2018)). Our results further expand this consensus by demonstrating CRYAB and HSP27 are dependent upon each other, but also have potentially different influences in breast cancer. Our data suggest that ER and PR are likely to be key clients within the cancer epichaperome for HSP27. Indeed, the third cluster we identified likely includes the TNBC (ER^−^/PR^−^/HER2^−^) patient group with elevated CRYAB expression and reduced HSP27 expression (Fig. 2B and Table 2). Our assessment of the variable expression of HSP27 and CRYAB in the TMA24 is that each sHSP and their combinations affect the breast cancer epichaperome differently. This is supported by the statistically significant inverse relationship we report for key breast cancer clinical-pathological parameters (Table 2).

The recent literature using cluster-based analyses presents conflicting conclusions regarding the prognostic value of both HSP27, compared to its association with the luminal A group, and CRYAB and its association with the basal /TNBC group (Buttacavoli et al. 2021)(Ivanov et al. 2008; Moyano et al. 2006; Sitterding et al. 2008; Voduc et al. 2015; Zoppino et al. 2018). This issue is further compounded when datasets from patient cohorts with variable ER, PR status (Ősz et al. 2021) and treatments are used for analysis (http://kmplot.com/analysis/; (Lánczky and Győrffy 2021)). Our approach was to examine the variance of *HSP27* and *CRYAB* expression within the breast cancer population. Our cluster analysis of the TCGA dataset was a targeted approach considering the combination of *HSP27* and *CRYAB* expression given that these two sHSPs are coexpressed and will coassemble (Shatov et al. 2020; Zantema et al. 1992). This will affect their client selection and therefore their chaperone function (Arrigo and Gibert 2014; Mymrikov et al. 2020).

Our survival analyses are limited to a small TMA dataset, but they illustrate how protein co-expression and phosphorylation status can influence the conclusions drawn. We can generate three different conclusions regarding patient survival by changing the population phenotype. In the TMA24 dataset, CRYAB is a non-significant result in the population as a whole (Fig. 4A), is a marker for good prognosis in a TP53 WT population (Fig. 4B) and a marker for poor prognosis in the TP53 mutant population (Fig. 4C). Equally, HSP27 was not significant in the population as a whole, but the staining for HSP27-82pS then delivered a significant result (Fig. 4D).

*TP53* mutation status is important as demonstrated by our analysis of the unbiased breast cancer cohort represented in TMA24 (Fig. 4B, C). We suggest this key cell cycle regulator is a client within the breast cancer epichaperome for CRYAB (Table 2). It has been reported to regulate TP53 (Bai et al. 2003; Jin et al. 2009). *CRYAB* is also one of the most over-expressed genes associated with *TP53* mutations in the TCGA (http://ualcan.path.uab.edu/; (Chandrashekar et al. 2017)) in agreement with our data (Table 2). A quarter of breast cancers express mutant TP53 (Olivier et al. 2010). *CRYAB*, like *HSP27*, is a TP53 responsive gene (Liu et al. 2007; Watanabe et al. 2009) and it is striking that the expression of WT *TP53* is associated with high levels of HSP27, but low levels of CRYAB (Table 2). *TP53* mutations are associated with high expression of CRYAB, but not HSP27 (Table 2). From our analyses, HSP27 rather than CRYAB is more strongly associated with ER and PR steroid receptor expression, a conclusion supported by recent studies eg (Győrffy 2021) including those applying cluster analysis (Buttacavoli et al. 2021).

HSP27 phosphorylation affects both its oligomerization and its epichaperome (Arrigo 2017, 2018; Arrigo and Gibert 2012, 2013; Jovcevski et al. 2017; Peschek et al. 2013). It is noteworthy that other studies have reported that HSP27-78pS is associated with poor patient survival and is Her2 mediated (Hwang et al. 2020; Zhang et al. 2007). In our study, HSP27-82pS (Fig. 4D) is also associated with better patient survival within the HSP27 cohort of the unbiased TMA24 dataset. IHC analyses are recognized as a gold standard in clinical practice despite the quantification and multiplexing limitations (Ősz et al. 2021) and therefore this reagent should prove useful for future breast cancer studies. Equally, our data on patient survival indicate caution is needed when the analyses of population studies do not take into account protein complex formation especially when there are potential functional consequences as in the case of HSP27 and CRYAB (Arrigo 2018; Arrigo and Gibert 2013, 2014; Mymrikov et al. 2020). This study could be the first of many that unpicks previously contradictory conclusions in relation to the role and impact of HSP27 and CRYAB in breast cancer. It aligns HSP27 and CRYAB to breast cancer patient groups based on their ER/PR and HER2 expression characteristics which are key clinical-pathological parameters in patient prognosis.

## Abbreviations

Akt: Protein kinase B
AN: Anova test
CRYAB: αB-crystallin or HSPB5
DAB: Diaminobenzidine
DMFS: Distant metastasis-free survival
ER: Oestrogen receptor
FET: Fishers Exact Test
IHC: Immunohistochemistry
HER2: Human epidermal growth factor receptor 2
HSP: Heat shock protein
HSP27-15pS: Phosphorylated HSP27 at serine 15
HSP27-78pS: Phosphorylated HSP27 at serine 78
HSP27-82pS: Phosphorylated HSP27 at serine 82
LR: Log Rank
MAPK: Mitogen-activated protein kinase
PR: Progesterone receptor
SCF: Skp, Cullin, F-box containing complex
sHSP: Small heat shock protein
TBS: Tris-buffered saline
TMA: Tissue microarray
TNBC: Triple negative breast cancer
TP53: Tumor protein p53
WT: Wild type
